# Supplementary material for: Development of Nonstructural Protein-Based Indirect ELISA to Identify Elephant Endotheliotropic Herpesvirus (EEHV) Infection in Asian Elephants (Elephas maximus)
Source: Animals (Basel). 2022 Jul 7;12(14):1747. doi: 10.3390/ani12141747 (PMC9312089; doi:10.3390/ani12141747)
Supplement: Supplementary file 1 [file animals-12-01747-s001.zip › animals-1785138 - Supplementary.pdf]

animals

Supplementary Tables

Table S1. Summary of the primers used in this study.

Genes	Sequence (5′-3′)	Size (bp)	References
Nested PAN-EEHV polymerase	Forward: ACAAACACGCTGTCRGTRTCYCCRTA	500	Latimer, 2011
	Reverse: GTATTTGATTTYGCNAGYYTG-TAYCC		
	Forward: ACAAACACGCTGTCRGTRTCYCCRTA	250	
	Reverse: TGYAAYGCCGTNTAYGGATTYAC-CGG		
EEHV1A Terminase	Forward: GTACGTCCTTTCTAGCTCAC	337	Richman, 1999
	Reverse: GTGTCGGCTAAATGTTCTTG		
EEHV4 Terminase	Forward: GTGCTGTAGCGGATCATGTC	310	Garner, 2009
	Reverse: CGTGCAACACGAGCACGCAAAG		

Table S2. Checkerboard titration of antigen and HRP-conjugated recombinant protein G. Bold numbers are indicative of the optimal concentrations for ELISA.

Antigens	Concentration of Antigens (μg/mL)	Dilution of HRP-Conjugated Recombinant Protein G		
		1:1000	1:1500	1:2500
EEHV-DNApol	30	3.125	3.068	2.992
	**15**	3.608	**3.5**	3.395
	7.5	3.318	3.318	3.096
	3.75	3.317	3.43	3.339

Table S3. Detection of EEHV antibodies using the EEHV-DNApol ELISA in various groups of elephant sera.

Group	Sample Amount	Seropositive Sample	OD_{450} Range	average OD_{450}	SD
A	14	0	0.565-1.756	1.038	0.368
B	4	0	0.759-1.436	0.996	0.301
C	150	21	0.086-3.456	1.340	0.639
D	7	0	0.124-2.121	0.556	0.714
**Total**	175	21			
